# Aspirin in the Form of Microneedle Repairs DNA and Reduces Inflammation in Persistent Skin Damage

**DOI:** 10.34133/bmr.0083

**Published:** 2024-09-16

**Authors:** Wenbin Cao, Huanchun Xing, Shuai Guo, Lin Wang, Xin Sui, Lijuan Huang, Yuan Luo, Jun Yang, Yongan Wang

**Affiliations:** ^1^ State Key Laboratory of Toxicology and Medical Countermeasures, Beijing Institutes of Pharmacology and Toxicology, Beijing 100850, China.; ^2^ Tianjin University of Science and Technology, Tianjin 300222, China.; ^3^ Hebei University of Science and Technology, Shijiazhuang 050018, China.; ^4^ Department of Bacteriology, Capital Institute of Pediatrics, Beijing 100020, China.

## Abstract

Skin damage caused by chemical corrosion is currently one of the common skin diseases and poisoning symptoms, with nitrogen mustard compounds causing the most persistent and severe damage. These chemicals penetrate the top layer of the skin, enter the dermis, and cause DNA damage, oxidative stress, and inflammation. However, to date, no effective drug treatment has been found. Even the potential antidotes could not effectively penetrate the top layer of the skin to exert their effects due to the skin barrier. To address this problem, an innovative transdermal drug delivery strategy based on aspirin microneedles was proposed. The classic medicine aspirin was first discovered not only to reduce inflammation and oxidative stress but also to promote DNA repair and reduce DNA damage. The aspirin microneedles directly delivered the drug to the damaged area, released aspirin through the skin barrier, and exhibited good biocompatibility. These findings indicate that aspirin microneedles have great potential for promoting wound healing and broad application prospects.

## Introduction

Due to their ease of production and storage, high lethality, and broad contamination range, highly corrosive and toxic nitrogen mustard (NM) compounds [1], including bifunctional alkylating agents sulfur mustard (SM) [2] and 2-chloroethyl ethyl sulfide (CEES) [3], have been widely adopted by numerous countries as chemical warfare agents in the vesicant category and have recently been used in wars and terrorist attacks. Additionally, compounds such as cyclophosphamide [4] and isocyclophosphamide, as alkylating chemotherapeutic agents, are used with extreme caution due to their high toxicity [5]. However, due to their complex mechanisms of action and severe damaging effects, there has been no antidote for NM compounds to date.

NM, with its strong DNA cross-linking properties, primarily disrupted DNA replication [6] and hindered cell division, leading to severe toxicity. Designing an antidote to directly repair NM-damaged DNA presented substantial challenges, requiring the theoretical insertion of highly reactive functional groups into DNA to remove NM and restore its functionality. However, such a compound could be associated with significant safety risks; if the highly reactive functional groups from the candidate antidote erroneously bound to DNA sites instead of NM, it could further damage the DNA. Therefore, feasible solutions focused on indirectly achieving DNA repair by enhancing and activating proteins and enzymes to stimulate or accelerate the natural DNA repair capabilities of organisms. In addition, NM caused extensive damage to proteins, enzymes, and the nervous system [7], resulting in persistent injury. Current antidote only alleviated symptoms of inflammation. Hence, an ideal antidote should not only induce anti-inflammatory and antibacterial actions but also comprehensively target the DNA damage repair pathway.

In treating poisoning, the antidote needs to be pharmacologically effective against toxins and also accurately delivered and accumulated at the poisoning site. Therefore, constructing targeted drug delivery systems is also crucial for detoxification. Since NM can be stored in subcutaneous fat [8], penetrated deeply into the dermis [9], and spread into the bloodstream [7], developing antidote delivery systems remains challenging. Typically, drugs only superficially penetrate the skin when applied topically [10] and do not reach the dermis or other affected areas even when administered orally or intravenously. Thus, one of the challenges in treating skin injuries is ensuring effective delivery of the therapeutic agent across the skin barrier.

Aspirin, a versatile medication with a long history, offers a wide range of benefits in human healthcare by effectively preventing the production of prostaglandins through the inhibiton of cyclooxygenase (COX) enzymes [11], leading to reduced inflammation and alleviation of associated symptoms. By inhibiting platelet aggregation, aspirin is also widely used to prevent and treat heart diseases [12]. Additionally, aspirin demonstrated promising anticancer effects by hindering the secretion of prostaglandins from tumor cells, thereby preserving cellular and humoral immunity, and has the potential to repair radiation-induced DNA damege [13]. Therefore, aspirin could be a potential candidate for treating NM-induced injuries.

**Fig. 1.**
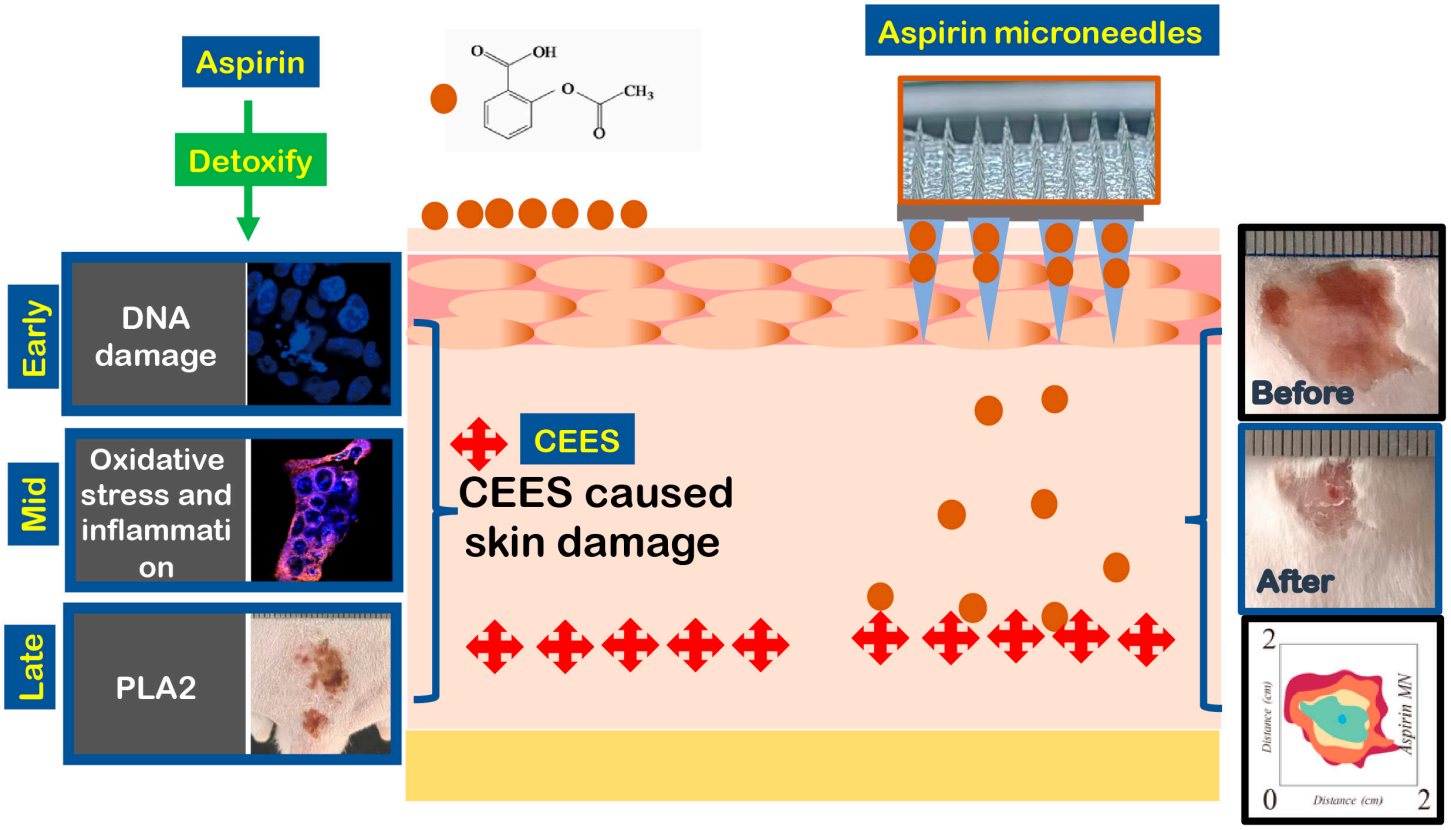
Schematic diagram of application for aspirin to repair NM-induced skin damage delivered by MNs.

Considering the complex requirements of NM detoxification and the broad application of aspirin, this study aimed to investigate whether aspirin can effectively treat initial DNA damage, subsequent inflammation [14], later phospholipase A2 (PLA2) stimulation, and other relevant factors. Additionally, the optimal form of therapeutic drug administration for skin injuries was explored, as shown in Fig. 1. The ultimate goal of this study was to discover an aspirin drug delivery system that achieved both temporal and spatial treatment coverage for detoxification.

## Materials and Methods

### Animals

All experiments were performed in accordance with the Regulations of the Experimental Animal Administration, issued by the State Committee of Science and Technology of the People’s Republic of China (1988 November 14), the ARRIVE Guidelines, and the Guidelines for the Care and Use of Laboratory Animals of the Beijing Institute of Pharmacology and Toxicology. The experiments were approved by the Animal Ethics Committee of the Beijing Institute of Pharmacology and Toxicology [Institutional Animal Care and Use Committee (IACUC) permit number: IACUC-DWZX-2023-P649]. BALB/c (19 ± 2 g) mice were obtained from Beijing Vital River Laboratory Animal Technology Co. Ltd. (Beijing, China). Rodents had free access to sterilized food and distilled water and were maintained in stainless steel cages filled with hardwood chips in an air-conditioned room on a 12:12 h light/dark cycle.

### Cell culture and treatment

HaCaT cells were obtained from the cell bank of Chinese Academy of Sciences. The cells were cultured in Dulbecco’s modified Eagle’s medium (DMEM), supplemented with 10% fetal bovine serum (FBS), penicillin (100 U/ml), and streptomycin (100 μg/ml). Cells were incubated at 37 ± 0.5 °C with 5% CO_2_ and 90% relative humidity and passaged every 2 to 3 d.

Further, to determine the effect of CEES on DNA, the expression of the DNA damage marker γH2AX was detected using a Western blot experiment after treating the cells with CEES (6 mM) for various durations (0.5, 1, and 24 h).

To investigate the effect of aspirin on repairing DNA damage, the expression of the damage marker γH2AX and repairing protein BRCA1 was detected. The cells were exposed to untreated medium, CEES (3 mM), CEES (3 mM) + aspirin (0.125 mM), and CEES (3 mM) + *N*-acetylcysteine (NAC; 0.125 mM) for 24 h, followed by a Western blot assay.

### In vitro cell viability study

The Cell Counting Kit-8 (CCK-8) assay was used to evaluate the cell viability of HaCaT cells treated with CEES, aspirin, NAC, CEES + aspirin, and CEES + NAC. The cells were seeded into 96-well plates at a density of 20,000 cells per well, cultured for 24 h to allow attachment, and then incubated with CEES (0.375, 0.75, 1.5, 3, and 6 mM), aspirin (0.125 mM), NAC (0.125 mM), CEES (3 mM) + aspirin (0.125 mM), and CEES (3 mM) + NAC (0.125 mM) for 24 h, respectively. Subsequently, the cells were treated with CCK-8 for 2 h at 37 °C. Six replicates were used in each case. The absorbance was measured at 450 nm using a microplate reader.

### Cell PI staining

Membrane integrity was assessed using propidium iodide (PI) staining to investigate the effects of PLA2 + 1-palmitoyl-2-oleoyl-sn-glycero-3-phosphoserine (POPS) and 1-palmitoyl-2-hydroxy-sn-glycero-3-phospho-L-serine (LPS; 16:0) on membrane permeability. Cells were incubated for 10 min with the following materials at different concentrations: PLA2 (0.1 and 1 U/ml), POPS (800 μg/ml), LPS 16:0 (0.05 and 0.5 μg/ml), and PLA2 + POPS (0.1 U/ml + 800 μg/ml and 1 U/ml + 800 μg/ml). The cells were then stained with PI (50 μg/ml) and Hoechst 33342 and imaged via laser scanning confocal microscope (LSCM).

To investigate the effect of CEES on apoptosis, PI staining and calcein-AM staining were simultaneously employed. Cells were incubated with CEES for different durations (0.5, 1, 2, 4, 8, 12, and 24 h), stained with PI (50 μg/ml), calcein-AM, and Hoechst 33342, and then imaged via LSCM.

### Fabrication of the Aspirin@HA-MNs

Hyaluronic acid (HA) and aspirin were mixed in phosphate-buffered saline. Then, 500 μl of the mixed solution was filled into a mold and centrifuged (3,000 rpm, 5 min) to infill the microneedle (MN) pinholes. Polyvinyl alcohol (PVA) (15%, m/m) was subsequently added to fill the mold to form the base of MN. Finally, the shaped Aspirin@HA-MNs were removed from the mold.

### Micronuclei detection

To investigate the therapeutic effect of aspirin against CEES, the micronucleus formation of cells was evaluated. Cells were poisined with CEES and treated with aspirin for different durations (0.5, 1, 2, 4, 8, 12, and 24 h). Subsequently, the cells were stained with Hoechst 33342 and imaged via LSCM. The nuclei were imaged and marked with fragments to reflect the DNA damage.

### JC-1 staining

HaCaT cells were seeded on a confocal dish and treated with CEES (3 mM), CEES (3 mM) + aspirin (0.125 mM), and CEES (3 mM) + NAC (0.125 mM) for 1 h. After treatment, HaCaT cells were incubated with 1 ml of JC-1 dye (20 μM) at 37 °C for 15 min and then imaged via LSCM.

### Fluorescein in skin

Kunming mice were divided into 10 groups and administered drugs as follows: hydrophilic cy3 via tail vein injection (1 and 10 mg/ml), hydrophobic cy3 via tail vein injection (1 and 10 mg/ml), hydrophilic cy3 smearing to skin (1 and 10 mg/ml), hydrophobic cy3 smearing to skin (1 and 10 mg/ml), and cy3@MNs (1 and 10 mg/ml). One hour after administration, skin samples were collected, fixed in 4% paraformaldehyde, and observed pathologically with fluorescence.

### Western blot analysis

HaCaT cells and skin tissues were lysed using lysis buffer (1% protease inhibitor and phosphatase inhibitor). Protein quantification was performed using the bicinchoninic acid (BCA) kit. Proteins were separated by SDS-PAGE gel and transferred to polyvinylidene difluoride membrane by incubating with fast sealing solution for 20 min. The membranes were washed with tris-buffered saline (TBST) three times for 5 min each and then incubated with primary antibodies overnight at 4 °C. The blots were washed with TBST and incubated with the appropriate horseradish peroxidase-conjugated secondary antibodies for 1 h at 37 °C. After reaction, the samples were washed with TBST to remove the unreacted antibodies, developed with enhanced chemiluminesence substrate, and exposed to x-ray film. An actin antibody was used as a loading control.

### Evaluation of the survival time of poisoned animals

After hair-shaving on the mouse’s back, the treated skin was smeared with CEES (1, 5, 7, 10, and 15 μl) and then gently covered with a plastic film for 10 min to enhance absorption. The survival time of mice was recorded.

### Evaluation of treatment effect in vivo

Male mice weighing 18 to 22 g were used to evaluate the treatment effects of Aspirin@HA-MNs in wound healing. The mice were poisoned with 1.5 μl of CEES on the hair-shaved skin as described before and then treated with two MNs (Aspirin@HA-MNs and NAC@HA-MNs) 1 h after poisoning. On the 2nd, 4th, 6th, 8th, and 10th days, the wounds were observed and photographed. On the 10th day, the subcutaneously regenerated tissues were collected and examined.

### Safety evaluation of aspirin

Kunming mice were administered aspirin (1 and 10 mg/ml). The major organs and blood of the mice were collected 24 h later. The tissues were analyzed by histopathology. Blood samples were tested for biochemical indicators.

### Histopathology and immunohistochemistry

On the 10th day after CEES exposure, the poisoned skins were fixed in 4% paraformaldehyde, dehydrated, and embedded in paraffin. Each sample was cut into 4-μm-thick slices for further staining [hematoxylin and eosin (H&E), Masson, immunohistochemical, and immunofluorescence].

### Statistical analysis

Statistical analysis was performed using GraphPad Prism 8.0 software. All data shown in the histograms were the results of at least three independent experiments and were presented as the mean ± SEM or mean ± SD. The sample size (*n*) for each statistical analysis and statistical methods used to assess significant differences is indicated in the figure legends. Differences between values were considered statistically significant when *P < 0.05, **P < 0.01, ***P < 0.001, and ****P < 0.0001.

## Results

### Dose- and time-damaged effects of CEES in vivo and in vitro

**Fig. 2.**
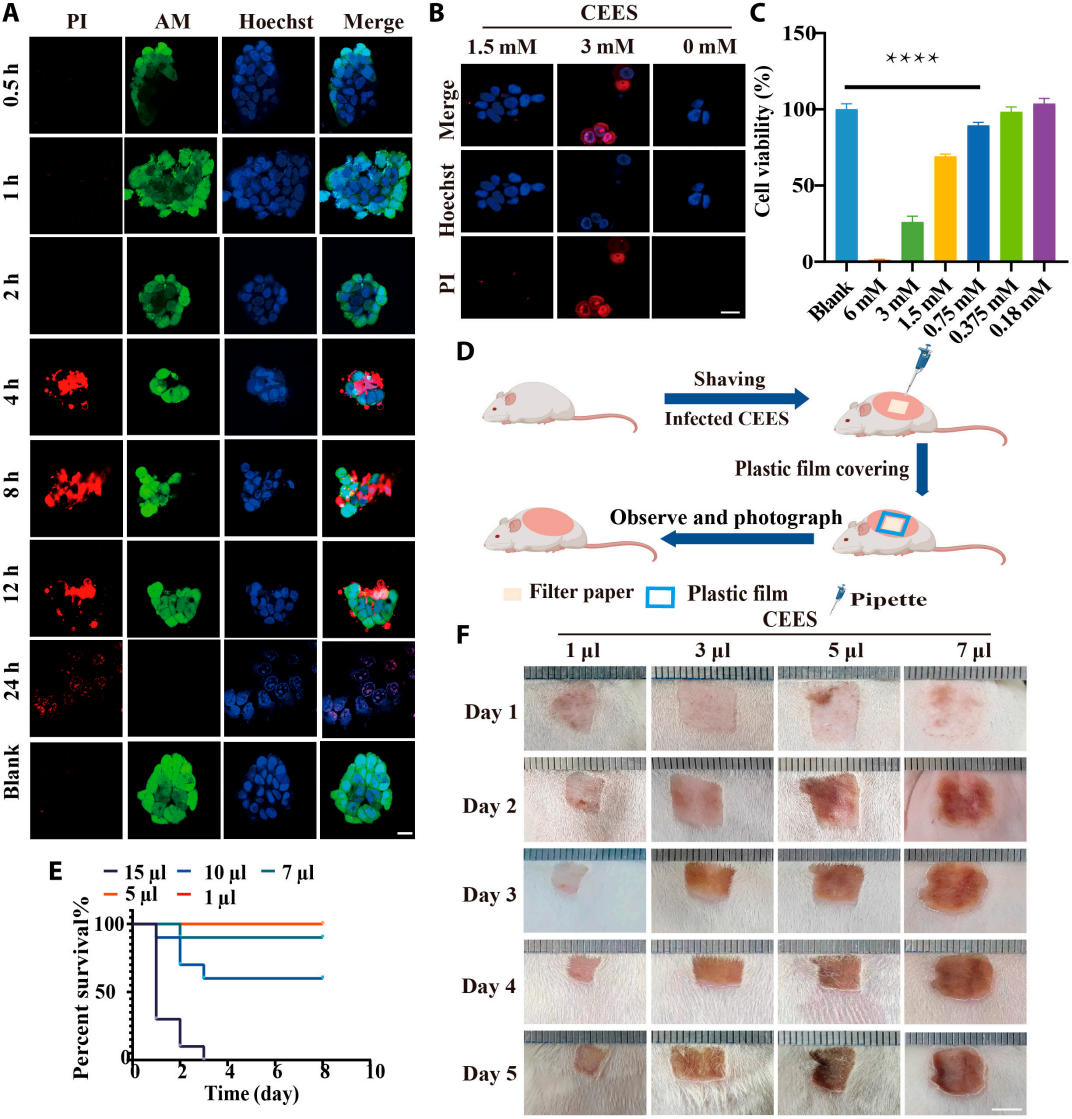
Dose- and time-damaged effect of CEES in vivo and in vitro. (A and B) LSCM fluorescent images of propidium iodide (PI)-stained CEES-treated cells at different times (0 to 24 h). (C) Viability of CEES-treated HaCaT cells. Data are presented as mean ± SD (*n* = 6). (D) Schematic illustration of the animal poisoning process. (E) Survival analysis of mice poisoned with CEES (1 to 15 μl). Data are presented as mean ± SD (*n* = 10). (F) Images of skin wounds developed on the mouse backs following CEES (1 to 7 μl) exposure. ****P < 0.0001.

First, we evaluated the dosage, duration, and symptoms of in vitro and in vivo CEES-induced damage. CEES (3 mM) stimulation of cells resulted in cellular apoptosis with cell membrane disruption within 4 h (Fig. 2A). However, if the concentration of CEES was reduced by half (1.5 mM), obvious cell apoptosis would not take place within 4 h (Fig. 2B). More specifically, approximately 70% of the cells were destroyed in 3 mM CEES after 24 h. When the concentration was below 0.75 mM, CEES would not cause significant cell apoptosis during the entire monitoring period (Fig. 2C). In animal studies, the traditional method of inducing skin poisoning was improved by applying a layer of plastic film to the skin after CEES exposure to prevent the evaporation of toxic compounds and enhance the permeation and absorption of toxins into the skin, resulting in more pronounced wound effects (Fig. 2D and Fig. S1). At a dosage of 5 μl of CEES, animals began to die within 48 h, and at 15 μl of CEES, 100% mortality was observed within 72 h (Fig. 2E). There were no evident wounds in the poisoned area within 24 h (Fig. 2F) when exposed in all concentrations. After 48 h, pronounced symptoms, such as redness and swelling [15], were observed. Within 76 h, the symptoms became severe, followed by the appearance of scabs. These findings suggested that CEES was highly toxic both in vitro and in vivo, causing severe latent damage.

### CEES-induced DNA damage

**Fig. 3.**
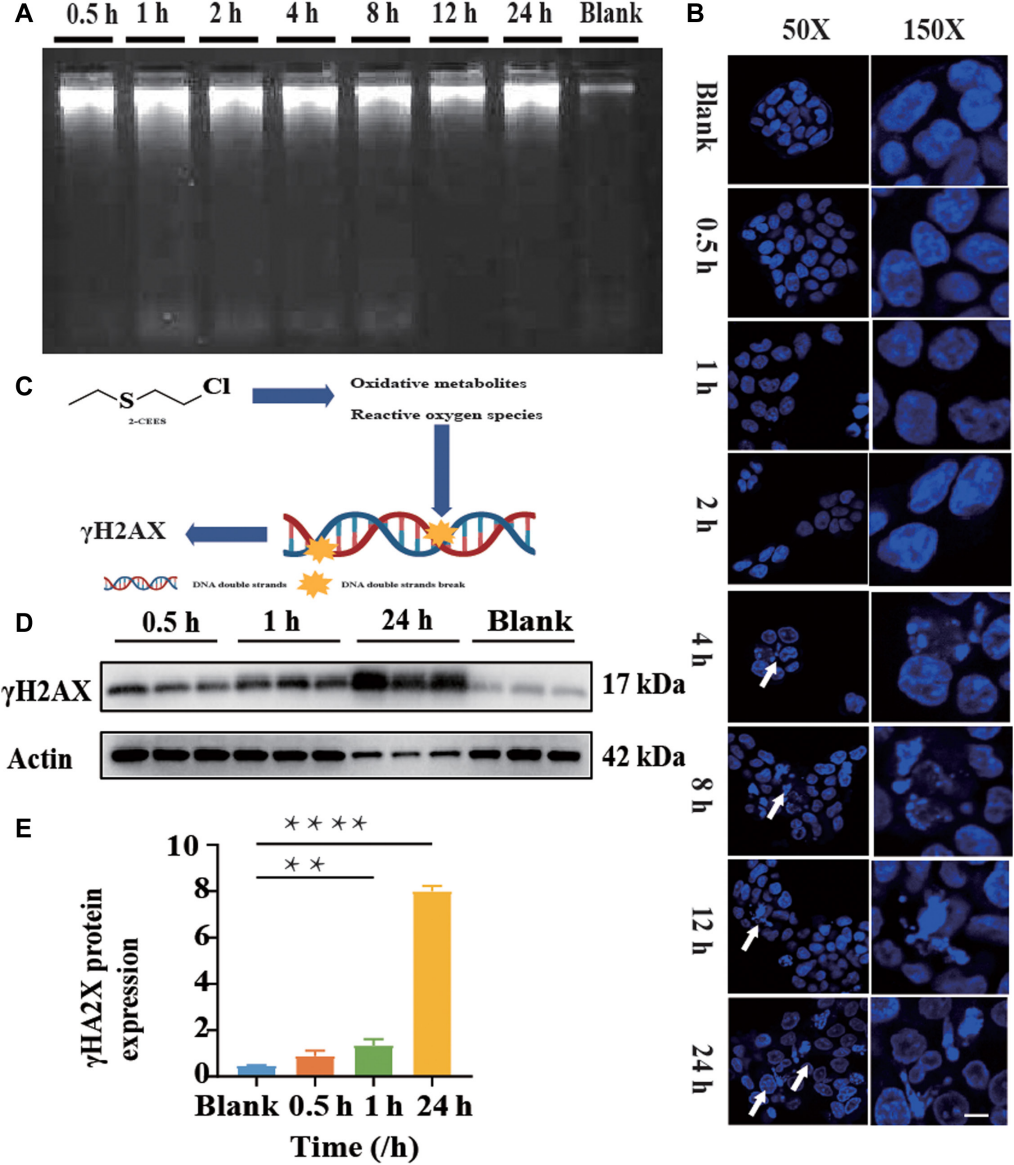
DNA damage caused by CEES. (A) DNA damage in CEES-treated cells at different times was detected by agarose gel electrophoresis. (B) LSCM images of micronuclei (indicated by arrowhead) in CEES (3 mM)-exposed HaCaT cells. (C) Schematic representation of γH2AX production in DNA damage. (D) Western blot of γH2AX protein expression in CEES (6 mM)-treated cells at different times. (E) Densitometric analysis of the Western blot data. Data are presented as mean ± SD (*n* = 3) and analyzed using the one-way analysis of variance (ANOVA) test. Significance of correlations was indicated by **P < 0.01, ****P < 0.0001.

NM primarily caused DNA damage via alkylation reactions, resulting in interstrand cross-links [16], thereby preventing efficient unwinding and other physiological processes, which resulted in nuclear fragmentation of the cell. In this study, 30 min following CEES exposure, DNA fragmentation was observed via gel electrophoresis (Fig. 3A), indicating that DNA cross-linking occurred in the cell nucleus. After 1 h, further DNA crosslinking triggered irregular cell nuclear division, as observed using the micronucleus assay (Fig. 3B). CEES directly disrupted the normal physiological structure of the nucleus [17]. At 30 min of CEES exposure, γH2AX (Fig. 3C), a sensitive biomarker for determining DNA damage and repair, increased significantly and continued to increase until 24 h. At 24 h, γH2AX expression was eightfold higher than that at 30 min (Fig. 3D and E). All data revealed that DNA damage occurred 30 min following CEES exposure and worsened with prolonged exposure. However, noticeable symptoms of damage, such as nuclear fragmentation, occurred after 4 h (Fig. 3B), indicating a lag period prior to the development of obvious poisoning-associated symptoms.

### CEES exposure triggered oxidative stress and inflammation

**Fig. 4.**
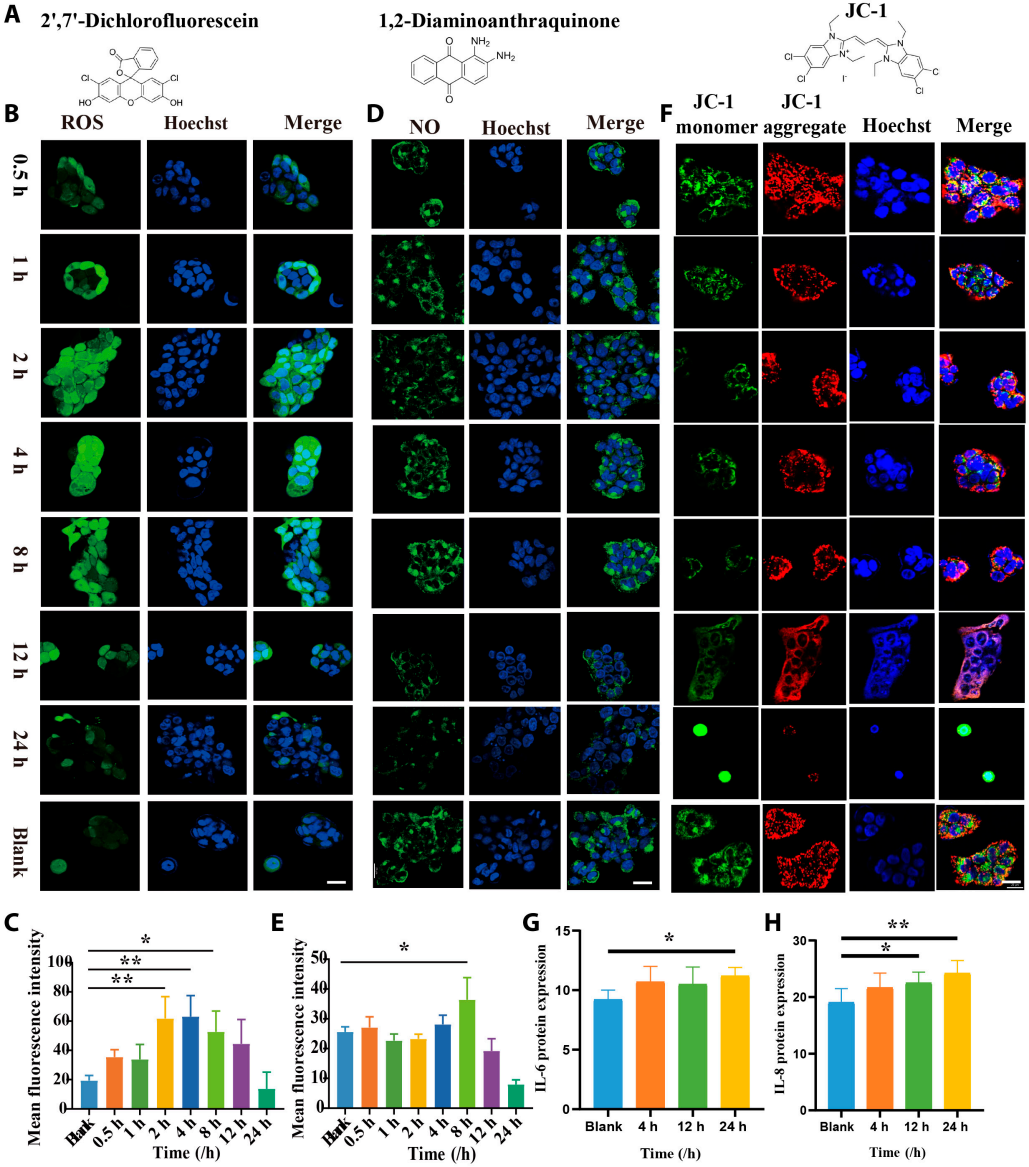
CEES exposure triggered oxidative stress and inflammation. (A) Different probe structures. LSCM showed ROS (B), NO (D), and mitochondrial membrane potential (F) changes in CEES exposure cells at different times. Densitometric analysis of the ROS (C) and NO (E) fluorescence data was performed. Data are presented as mean ± SD (*n* = 3). Enzyme-linked immunosorbent assay (ELISA) of IL-6 (G) and IL-8 (H) protein expression from cell supernatant of CEES-treated cells at different times was conducted. Data are presented as mean ± SD (*n* = 6) and analyzed using the one-way ANOVA test. Significance of correlations was indicated by *P < 0.05, **P < 0.01, ***P < 0.001.

NM’s strong alkylation ability can destroy crucial substances like proteins, nucleic acids, and enzymes, leading not only to the destruction of cell nucleus [17] but also to severe damage to other critical organelles within the cell, such as mitochondria, resuting in the disruption of mitochondrial function and triggering a series of reactions, including the excessive production of reactive oxygen species (ROS) [18]. Following CEES exposure, three probes (1,2-diaminoanthraquinone, JC-1, and dihydrofluorescein diacetate) were employed to monitor the expression of several key factors in cells within 24 h (Fig. 4A). ROS levels began to increase at 0.5 h, peaked at 2 h, and gradually decreased after 12 h (Fig. 4B and C). This trend was consistent with the changes in mitochondrial membrane potential (Fig. 4F). A significant decrease in mitochondrial membrane potential was observed 4 h after poisoning, indicating the onset of apoptosis in cells. At 12 h after poisoning, nitric oxide (NO) generation significantly decreased (Fig. 4D and E). Further investigation into inflammatory markers revealed a considerable delay. Secretion of IL-6 and IL-8 increased significantly at 24 h after CEES exposure (Fig. 4G and H). The data above demonstrated that the series of inflammation response triggered by NM develops gradually following DNA damage.

### CEES exposure induced an increase in PLA2

**Fig. 5.**
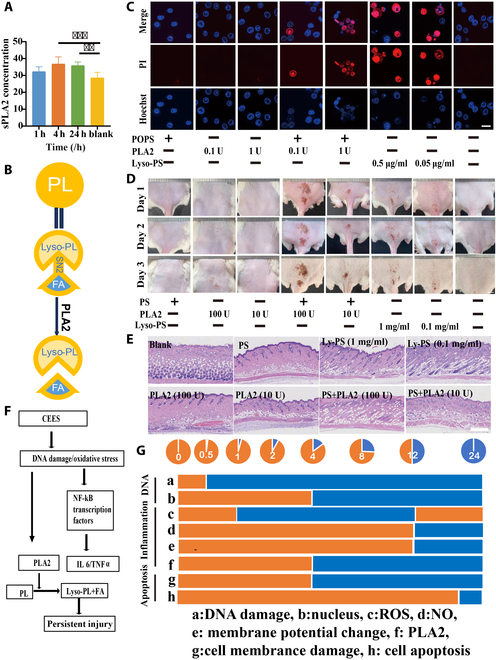
CEES exposure increased phospholipase A2 (PLA2). (A) ELISA of PLA2 protein expression in CEES-treated cells. Data are presented as mean ± SD (*n* = 3). (B) Schematic illustration of PLs converted into lysophospholipids and fatty acids by PLA2 catalytic decomposition. (C) Effects of different PLA2 combination drugs on cell membrane permeability. (D) Photos of skin damage caused by different PLA2 combinations of drugs. (E) Images showing hematoxylin and eosin (H&E)-stained skin sections from the groups treated with different PLA2 combination drugs. (F) Schematic diagram of PLA2 inflammatory pathways. (G) Schematic diagram of damage time effect of CEES. **P < 0.01, ***P < 0.001.

The relationship between CEES and PLA2 was not well understood. Following CEES exposure, PLA2 levels in the injured skin began to increase after 1 h, peaking at 4 h, and retained a statistically significant difference compared to the control group even at 24 h (Fig. 5A). As illustrated in Fig. 5B, PLA2 decomposed phospholipids (PLs) from the cell membranes into fatty acids, such as arachidonic acid, which further promoted inflammation as reported. However, another degradation product of PL, lysophosphatide (Ly-PL), was more likely to cause skin ulceration than arachidonic acid [19] but had received little attention. The damage caused by Ly-PL and PLA2 was evaluated in vivo through subcutaneous injection and in vitro in cells using PI staining. No skin ulceration or apoptosis occurred when PLA2 or phosphatidylserine (PS), a type of PL, was administered individually (Fig. 5C to E). However, when PLA2 was mixed with 1-palmitoyl-2-oleoyl-sn-glycero-3-phospho-L-serine (POPS), a type of the PS in membrance, to generate Lyso-POPS, skin ulceration and cell apoptosis occurred; the effects were similar to those observed with direct administration of 1-palmitoyl-2-hydroxy-sn-glycero-3-phospho-L-serine (Lyso-PS). These findings partially explained the underlying reason for the persistent skin ulceration caused by CEES.

Based on the cell injury data, a preliminary understanding of the CEES-induced cellular damage process has been obtained (Fig. 5F). Following CEES exposure, DNA breaks and ROS production occurred immediately, resulting in a decrease in mitochondrial membrane potential. Subsequently, nuclear fragmentation symptoms appeared at 4 h, and PI staining increased significantly, indicating the onset of apoptosis [20]. At 12 h, the expression of the inflammatory factor, NO, decreased, whereas the secretion of PLA2 increased significantly, occurring earlier than anticipated. The PLA2-catalyzed production of Ly-PL further exacerbated skin erosion. The inflammatory symptoms became evident 24 h later. In summary, the symptoms of DNA damage appeared earlier than those of inflammation, and the symptoms caused by damage manifested after a significant lag period (Fig. 5G).

### Aspirin fought oxidative stress and inflammation

**Fig. 6.**
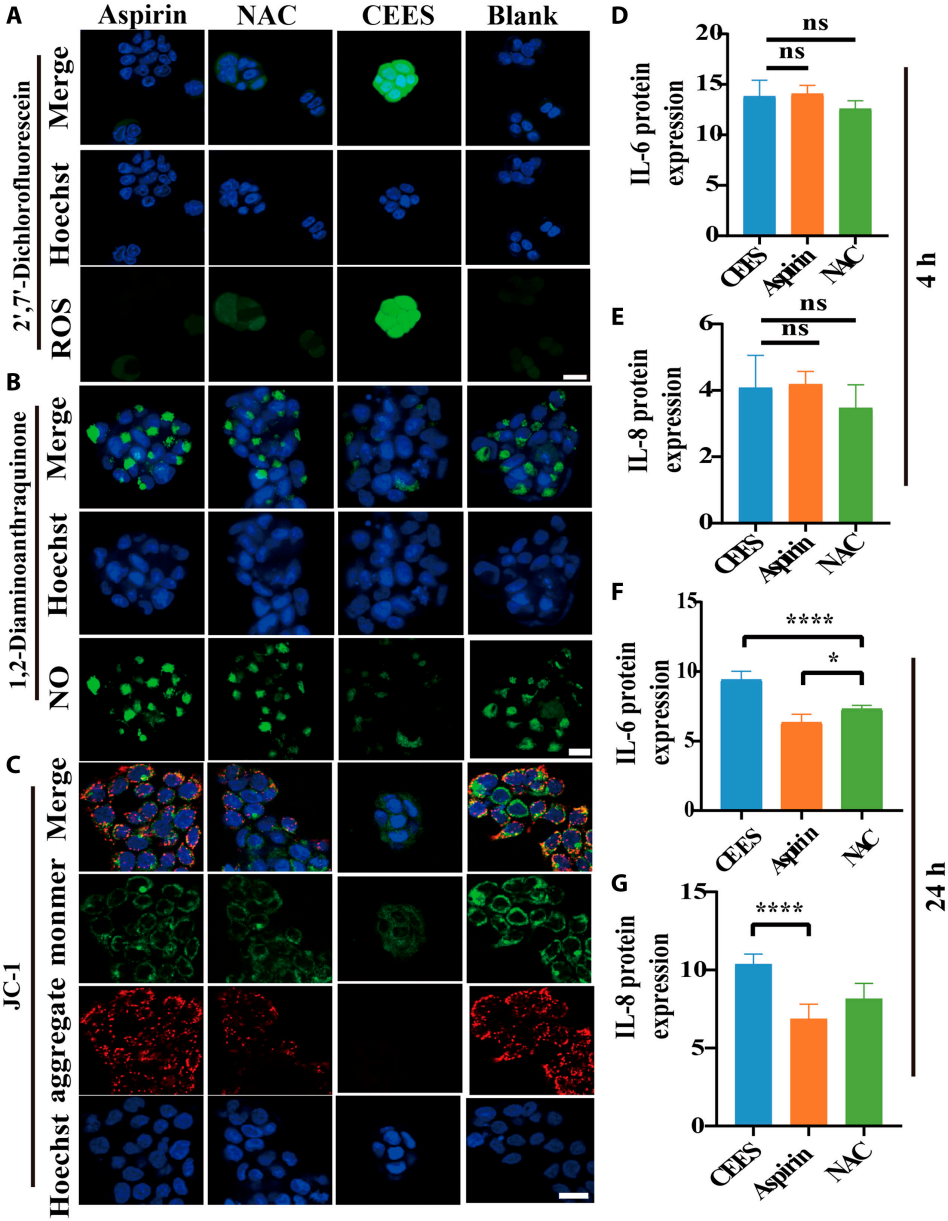
Aspirin alleviated oxidative stress and inflammation. LSCM images of ROS probes (A), NO probes (B), and JC-1 probes (C) in different administration groups after CEES exposure after 1, 12, and 4 h, respectively. ELISA of IL-6 (D) and IL-8 (E) protein expression from cell supernatant treated with CEES at 4 h. Data are presented as mean ± SD (*n* = 6). ELISA of IL-6 (F) and IL-8 (G) protein expression from cell supernatant treated with CEES at 24 h. Data are presented as mean ± SD (*n* = 6) and analyzed using the one-way ANOVA test. Significance of correlations was indicated by *P < 0.05, ****P < 0.001, ns: not significant.

Initially, the anti-inflammatory and anti-oxidative stress effects of aspirin were evaluated at the cellular level. Aspirin effectively inhibited the production of oxidative stress, as reflected by the fluorescence signals (Fig. 6). In the aspirin group, ROS production decreased after 1 h of exposure to 3 mM CEES (Fig. 6A), indicating a reduction in cellular oxidative stress. At 12 h, aspirin enhanced NO production [21], suggesting that aspirin may have promoted cell growth to mitigate damage (Fig. 6B). Additionally, the JC-1 probe showed that the membrane potential of the mitochondria increased in the aspirin-treated group after 4 h of exposure to 3 mM CEES, suggesting that aspirin may have a protective effect on the mitochondria, promoting normal energy metabolism and reducing apoptosis (Fig. 6C). Four hours after exposure to CEES, no significant expression of inflammatory markers was detected. This suggested that inflammation was not triggered during the initial phase of exposure (Fig. 6D and E). Due to the decrease in indicators such as ROS when treated with asprin, there was noticeable inhibition of cellular inflammation after 24 h, as indicated by the significant reduction in the levels of inflammatory markers IL-6 and IL-8 in the cell supernatant (Fig. 6F and G). Aspirin decreased ROS production, increased NO production, and reduced inflammation. Overall, aspirin demonstrated superior therapeutic effects compared to the positive control drug NAC [22].

### Aspirin worked against CEES at the cellular level

**Fig. 7.**
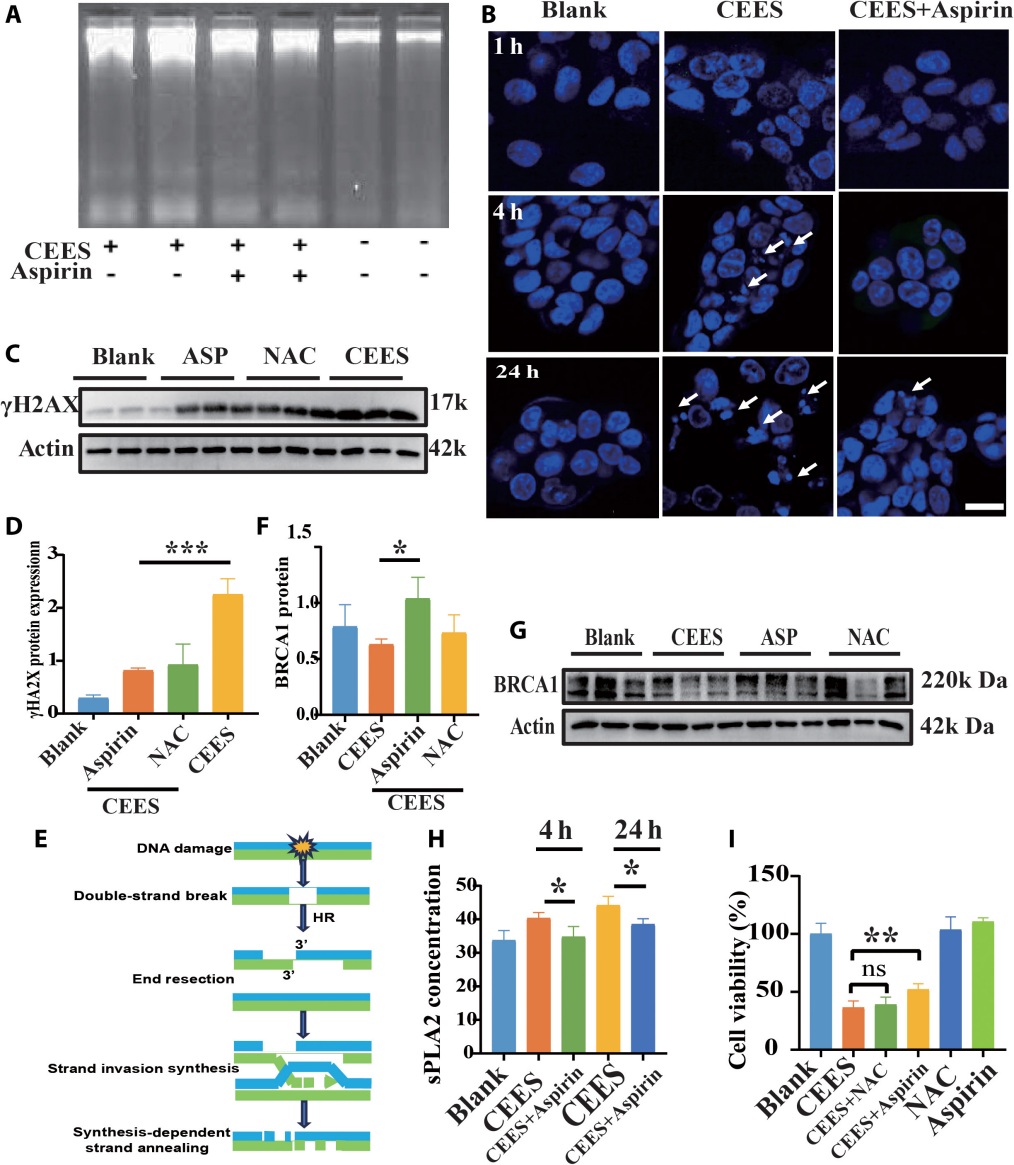
Aspirin inhibited the effects of CEES at the cellular level. (A) DNA repair by aspirin was detected by agarose gel electrophoresis. (B) LSCM of micronuclei (indicated by arrowhead) in CEES (3 mM)-exposed HaCaT cells treated with aspirin. (C) Western blot detected the expression of γH2AX protein in CEES- and aspirin-treated cells and densitometric analysis of the data (D). Data are presented as mean ± SD (*n* = 3). (E) Schematic diagram of homologous recombination repair. (G) Western blot detected the expression of BRCA1 protein in CEES-, aspirin-, and NAC-treated cells and densitometric analysis of the data (F). (H) The expression of PLA2 proteins in CEES- or aspirin-treated cell supernatants was detected by ELISA at different time durations. (I) Viability of HaCaT cells treated with NAC, aspirin, and CEES treated with aspirin or NAC. Data are presented as mean ± SD (*n* = 6) and analyzed using the one-way ANOVA test. Significance of correlations was indicated by *P < 0.05, **P < 0.01, ***P < 0.001.

In addition to its well-known anti-inflammatory effects, aspirin exhibited other novel functions, including DNA repair [23] and inhibition of PLA2 production in this work. Besides the damage caused by radiation-induced DNA breaks reported previously [13], this study also demonstrated that aspirin provided a significant repair effect against DNA cross-linking damage induced by CEES. Aspirin effectively prevented DNA fragmentation 0.5 h after exposure, indicating a protective effect (Fig. 7A), and further decreased nuclear fragmentation within 24 h, promoting normal cell mitosis progression (Fig. 7B). Further evaluation demonstrated a significant reduction in the expression of the DNA damage marker, γH2AX protein, following the administration of aspirin (Fig. 7C and D). Simultaneously, in the aspirin-treated group, aspirin repaired DNA through homologous recombination (Fig. 7E). BRCA1 protein expression, which enhanced DNA repair [24], increased significantly (Fig. 7F and G). These findings, based on the principle of homologous DNA recombination, strongly suggested that aspirin was effective in reducing DNA damage and promoting DNA repair. In contrast, NAC, a drug commonly used to combat CEES [22], did not significantly increase DNA repair proteins, revealing its limited DNA repair capabilities (Fig. 7F and G). The impressive DNA repair capabilities of aspirin made it a potentially effective agent against damage caused by NM.

The inhibitory effect of aspirin on PLA2 expression was also examined. The results clearly showed that aspirin significantly hindered inflammation, thereby further suppressing excessive PLA2 expression following inflammation (Fig. 7H). The efficacy of aspirin in enhancing the ability of cells to resist toxins was evaluated by survival rates using the CCK-8 assay. Aspirin treatment increased the survival rate of CEES-poisoned cells from 36% to 51%, whereas NAC showed no therapeutic effect (Fig. 7I). Importantly, aspirin administration did not result in any toxic effects at the cellular level (Fig. 7I). When the dual anti-inflammatory and DNA repair effects were simultaneously activated, aspirin exhibited a remarkable enhancement in cell survival, nearly doubling its efficacy.

### Effects of aspirin on CEES when differentially administrated

**Fig. 8.**
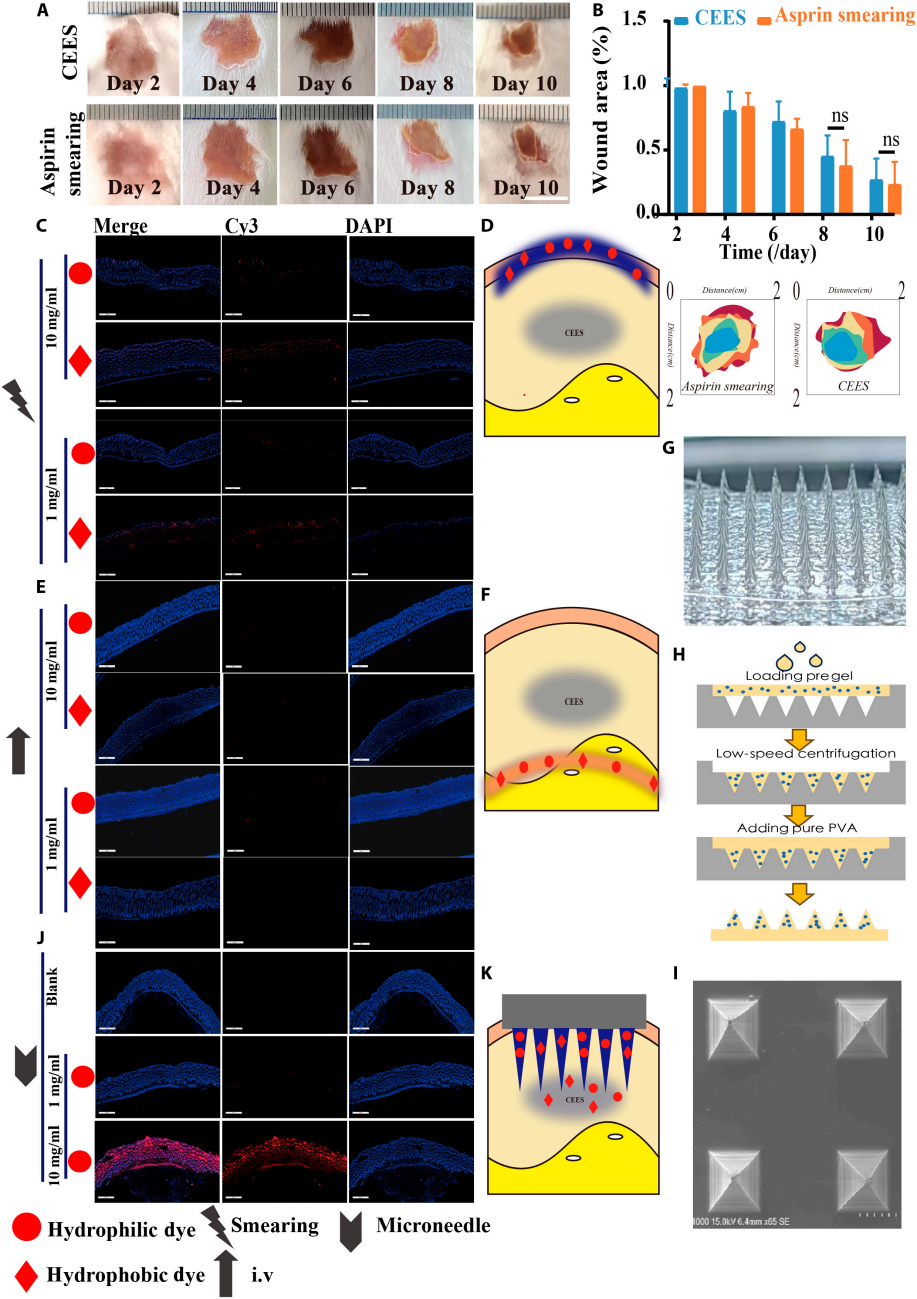
Effects of different administration modes of aspirin. (A) Photos of the skin wounds in different groups (control, liniment aspirin) within 10 d. (B) Quantitative analysis of the wound area in different groups (control, liniment aspirin). Data are presented as mean ± SD (*n* = 15). (C) Water- and fat-soluble dyes simulated drug skin administration. (D) Diagram of smearing dyes on skin. (E) Water- and fat-soluble dyes simulated intravenous drug administration. (F) Diagram of caudal vein administration. (G) Corresponding amplified optical images of MNs. (H) Schematic diagram of the MN fabrication process. (I) SEM images showing the microporous surface structure of the MNs. (J) MNs are equipped with water-soluble dyes to simulate the drug penetration process. (K) Schematic diagram of MN administration.

Once the potential of aspirin in treating CEES-induced damage was determined, further investigation was warranted. Although aspirin demonstrated positive therapeutic effects at the cellular level, these effects were not observed when applied topically to wounds in animal models (Fig. 8A and B). The primary reason for this outcomes was likely the inability of aspirin to effectively reach the site of CEES-induced damage. This result was closely related to the properties of both aspirin and CEES. Skin pathology experiments revealed that water-soluble dye-simulating medications adhered minimally to the outer layer of the skin when applied topically. Even with an increased dosage, the water-soluble dye could not penetrate the skin barrier (Fig. 8C and D). In contrast, lipid-soluble dyes simulating CEES could effectively penetrate the skin, whereas therapeutic medications could not (Fig. 8C and D). Furthermore, a previous study [25] demonstrated that animals developed scabs after CEES exposure, which further impeded aspirin’s ability to penetrate the affected sites. The location of the CEES injury was rather unique; therefore, even if the antidote was administered intravenously, the drugs still could not reach the injured area (Fig. 8E and F). Additionally, the phenomenon of NM being stored in subcutaneous fat highlighted the difficulty in effectively delivering the antidote to the site of CEES damage, contributing to the challenging and prolonged treatment of ulceration.

Consequently, soluble MNs loaded with aspirin were synthesized. Duo to their needle-like physical structure (Fig. 8G), they could forcibly penetrate the stratum corneum and effectively deliver the therapeutic drug aspirin to the affected area. The MNs were synthesized using a template method [26] (Fig. 8H), and scanning electron microscopy results showed that the MNs were 1 μm in height (Fig. 8I), meeting the requirements to penetrate the stratum corneum. Fluorescence pathological images following transdermal administration of MNs loaded with a fluorescent agent showed that the MNs successfully transported the loaded drug to the subcutaneous layer (Fig. 8J and K).

### Aspirin worked against CEES at the animal level

**Fig. 9.**
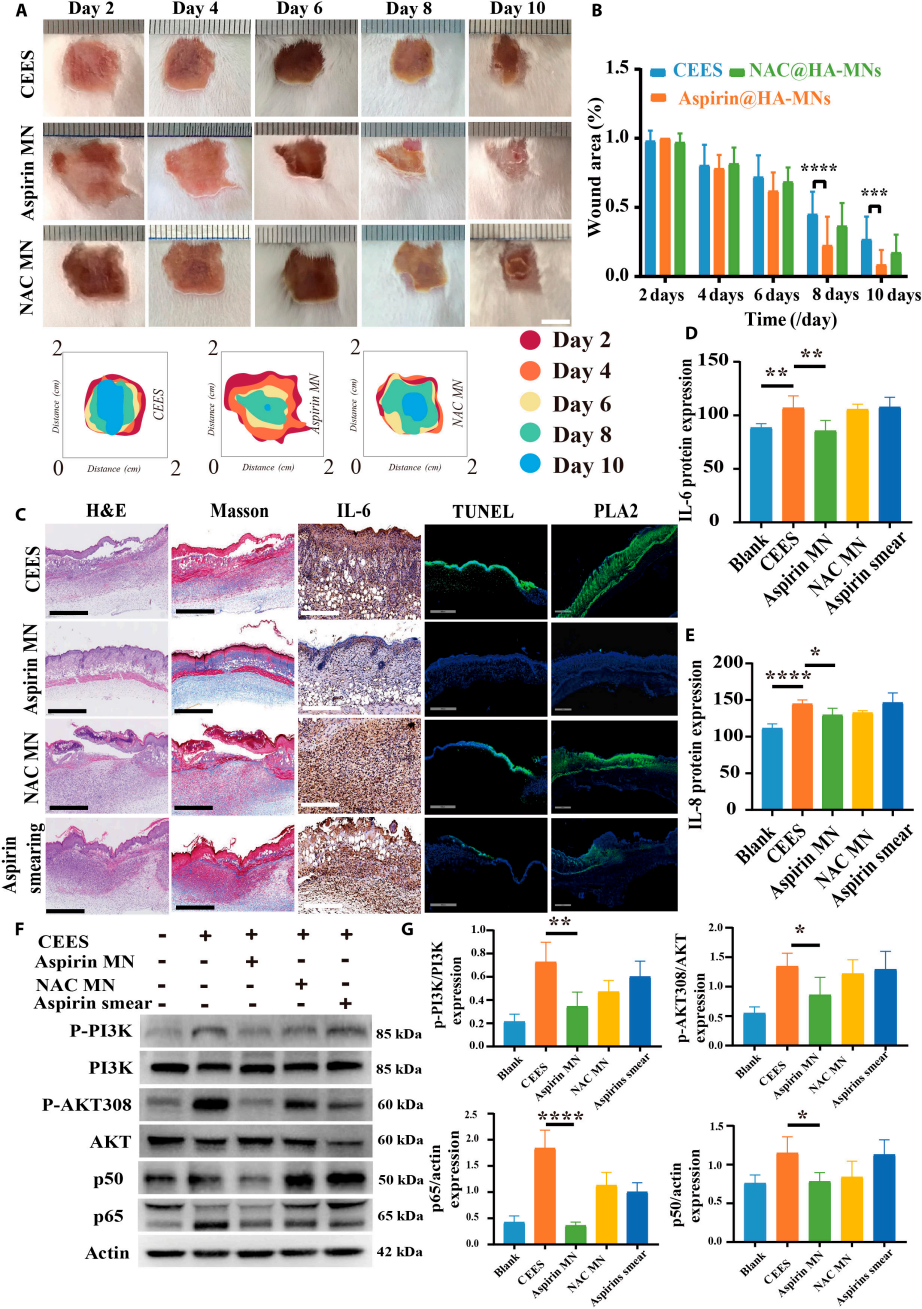
Aspirin worked against CEES at the animal level. (A) Photos (10 d) of the skin wounds in different groups (CEES, Aspirin MN, and NAC MN). (B) Quantitative analysis of the wound area in three groups (CEES, Aspirin MN, and NAC MN). Data are presented as mean ± SD (*n* = 15). (C) Pathological results of skin treated with antidotes. IL-6 (D) and IL-8 (E) expression in the animal skin, detected using ELISA. Data are presented as mean ± SD (*n* = 6). (F) Western blotting detected the effects of different administration groups on PI3K/AKT signaling pathway. (G) Densitometric analysis of the Western blot data. Data are presented as mean ± SD (*n* = 6). *P < 0.05, **P < 0.01, ****P < 0.0001.

A comprehensive and detailed evaluation of skin damage induced by NM was conducted using aspirin-loaded HA MNs (Aspirin MN). The area of the poisoned wounds at various time points indicated that after day 8, animals treated with Aspirin MN exhibited a significant reduction in wound area and better recovery, whereas there was no significant difference in the wound area between animals treated with NAC-MN, topical aspirin application, and the control (Fig. 9A and B). The pathological results indicated that following Aspirin MN treatment, the epidermal layer on the skin tissue surface recovered well, with no apparent damage, increased collagen deposition, decreased expression of IL-6, and reduced cell apoptosis, as demonstrated by the terminal deoxynucleotidyl transferase dUTP nick end labeling (TUNEL) assay results (Fig. 9C). Due to the effective inhibition of inflammation, PLA2 expression was reduced significantly, thereby decreasing the risk of continued interaction with cell membrane fragments and necrosis (Fig. 9C). Topical application in the aspirin group did not result in any significant improvement, which indirectly demonstrated that the damage induced by CEES was below the epidermis and required effective penetration of drugs into the epidermis. Merely performing superficial cleansing and anti-inflammatory treatments was insufficient for CEES treatment.

The mechanisms underlying the anti-inflammatory effects of aspirin in animal models were further investigated. Intially, second-generation sequencing of skin samples was conducted following CEES exposure. The repeatability of the samples was high, indicating reliability and credibility of the results obtained (Fig. S2A). The classical phosphatidylinositol 3-kinase (PI3K) pathway was examined using transcriptome analysis (Fig. S2B). After CEES stimulation, phosphorylated PI3K levels significantly increased, while the total expression of PI3K remained unchanged. This subsequently induced an increase in phosphorylated AKT downstream, resulting in the up-regulation of nuclear factor κB expression. Ultimately, this exacerbated inflammation and elevated the expression of interleukin-6 (IL-6) and IL-8 (Fig. 9D and E) in vivo. After the administration of Aspirin MNs, phosphorylated PI3K expression was effectively inhibited, leading to comprehensive down-regulation of phosphorylated AKT, p65, and p50 throughout the pathway, effectively suppressing inflammation (Fig. 9F and G).

### Safety of aspirin

**Fig. 10.**
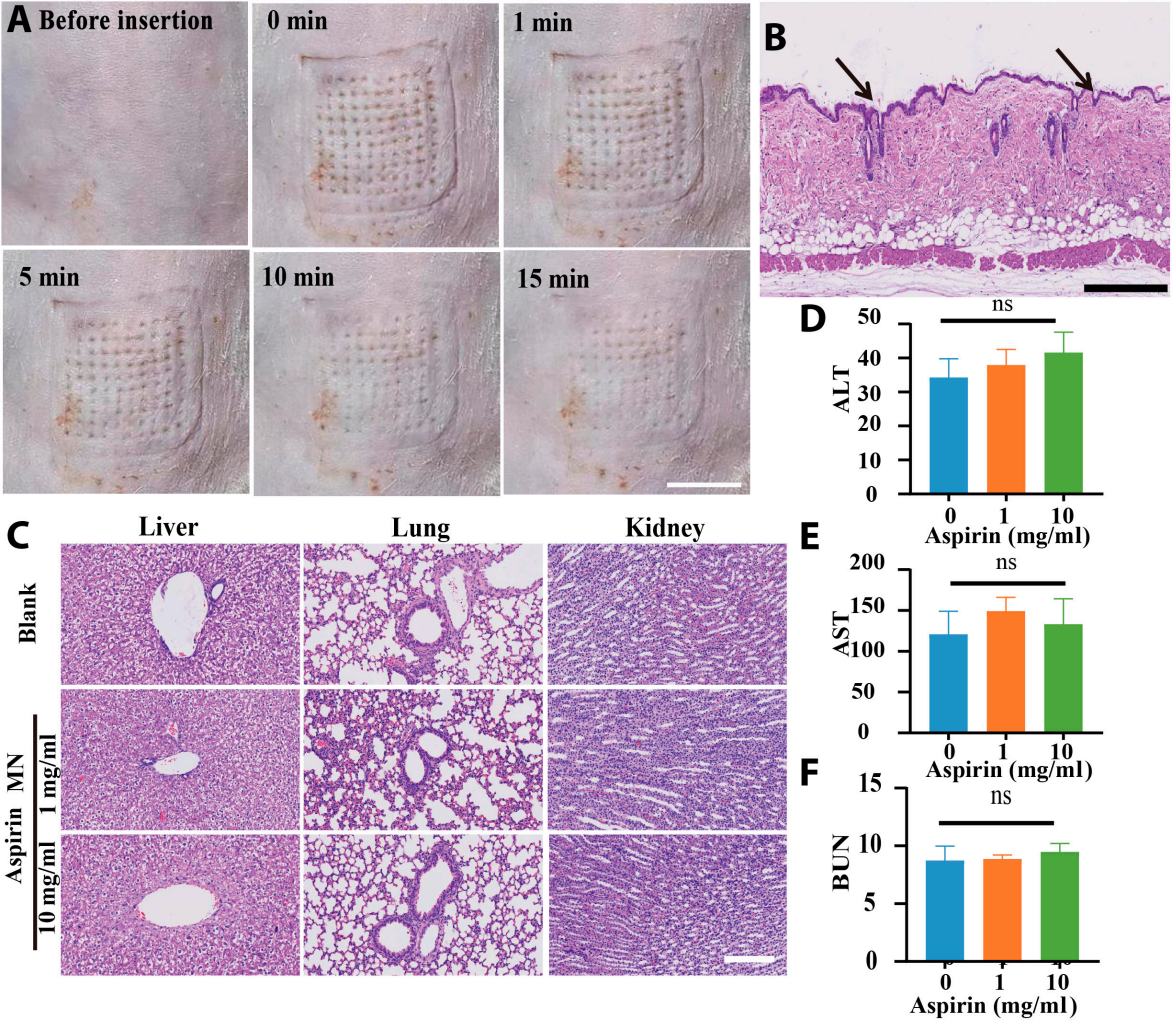
Safety of aspirin. (A) Safety after inserting blank MNs into skin. (B) Images of H&E-stained skin sections. (C) Images of H&E-stained liver, lung, and kidney sections. (D) Blood biochemistry of alanine transaminase (ALT), (E) aspartate transaminase (AST), and (F) blood urea nitrogen (BUN) in mice treated with aspirin. Data are presented as mean ± SD (*n* = 6).

Aspirin is a well-established medication with a long history of use, and its safety profile is well documented. This study focused on evaluating the safety of aspirin when delivered via MNs. Due to the use of soluble MNs, the needle puncture sites returned to normal 15 min after administration (Fig. 10A). Furthermore, no signs of inflammatory lesions were observed in the surrounding tissues at the site of the needle puncture (Fig. 10B). Additionally, the active ingredients loaded in the MNs did not significantly diffuse to major organs beyond the administration site (Fig. S3). This method of administration had the potential to improve patient compliance. Pathological findings confirmed that aspirin, delivered at safe concentrations via MNs, did not cause significant damage to vital organs (Fig. 10C). Moreover, blood biochemical measurements in the animal models remained within normal ranges (Fig. 10D to F). Notably, unlike traditional aspirin administration, no common side effects such as thrombolysis or liver damage were observed. In conclusion, the use of MN-delivered aspirin as well as the MN technology was determined to be relatively safe.

## Discussion

In this study, multiple targets and mechanisms of DNA damage, oxidative stress, inflammation, and subsequent PLA2-induced damage following CEES exposure were elucidated. Additionally, the well-established drug aspirin was empolyed to counteract the complex injury mechanisms induced by CEES toxins. At the cellular level, aspirin effectively addressed the entire course of injury, from initial DNA repair to the subsequent inhibition of oxidative stress, alleviation of inflammation, and reduction of PLA2 secretion in the later stages. The therapeutic effect of aspirin was superior to that of the standard control drug NAC. After identifying the therapeutic drug, further investigations at the animal level were necessary to determine the optimal method of adminstration. Aspirin exhibited no therapeutic effect when administered via traditional topical application. Consequently, MN technology was adopted. The aspirin-loaded MN effectively penetrated the epidermis, diffused to the subcutaneous area of the toxin-damaged site, and repaired the series of injuries induced by CEES. In conclusion, this study demonstrated that a well-established drug (aspirin) can be repurposed to expand its traditional use to effectively combat the global challenge of vesicant skin injuries caused by NM. Furthermore, aspirin demonstrated the ability to repair DNA damage induced by chemical agent. Additionally, the treatment principles for combating skin injuries were elucidated, emphasizing the importance of selecting drugs that address different injury mechanisms at various stages throughout the treatment timeline and adopting a rational drug delivery method to achieve accurate spatial distribution and effectively reach the affected areas. MN technology presented a promising approach for skin wound treatment.

## Data Availability

The data that support the findings of this study are available from the corresponding author upon reasonable request.
