# Supplementary material for: Aspirin in the Form of Microneedle Repairs DNA and Reduces Inflammation in Persistent Skin Damage
Source: Biomater Res. 2024 Sep 16;28:0083. doi: 10.34133/bmr.0083 (PMC11403356; doi:10.34133/bmr.0083)
Supplement: Supplementary 1 — Figs. S1 to S3 [file bmr.0083.f1.docx]

**Support information for**

**Aspirin in the Form of Microneedle Repairs DNA and Reduces Inflammation in Persistent Skin Damage**

**Wenbin Cao ^a^*^†^*, Huanchun Xing ^a, b^*^†^*, Shuai Guo ^a, c^, Lin Wang ^a^, Xin Sui ^a^, Lijuang Huang^a, d *^, Yuan Luo ^a^, Jun Yang ^a,*^ ,Yongan Wang ^a,*^**

a State Key Laboratory of Toxicology and Medical Countermeasures, Beijing Institutes of Pharmacology and Toxicology, Beijing, 100850, China

b Tianjin University of Science and Technology, Tianjin, 300222, China

c Hebei University of Science and Technology, Shijiazhuang, 050018, China

d Department of Bacteriology, Capital Institute of Pediatrics, Beijing 100020,China


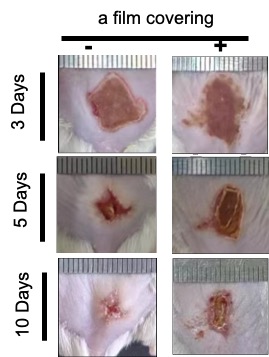


**Figure S1.** **Images of skin wounds** developed on the mice backs following CEES exposure with/without film covering.

***
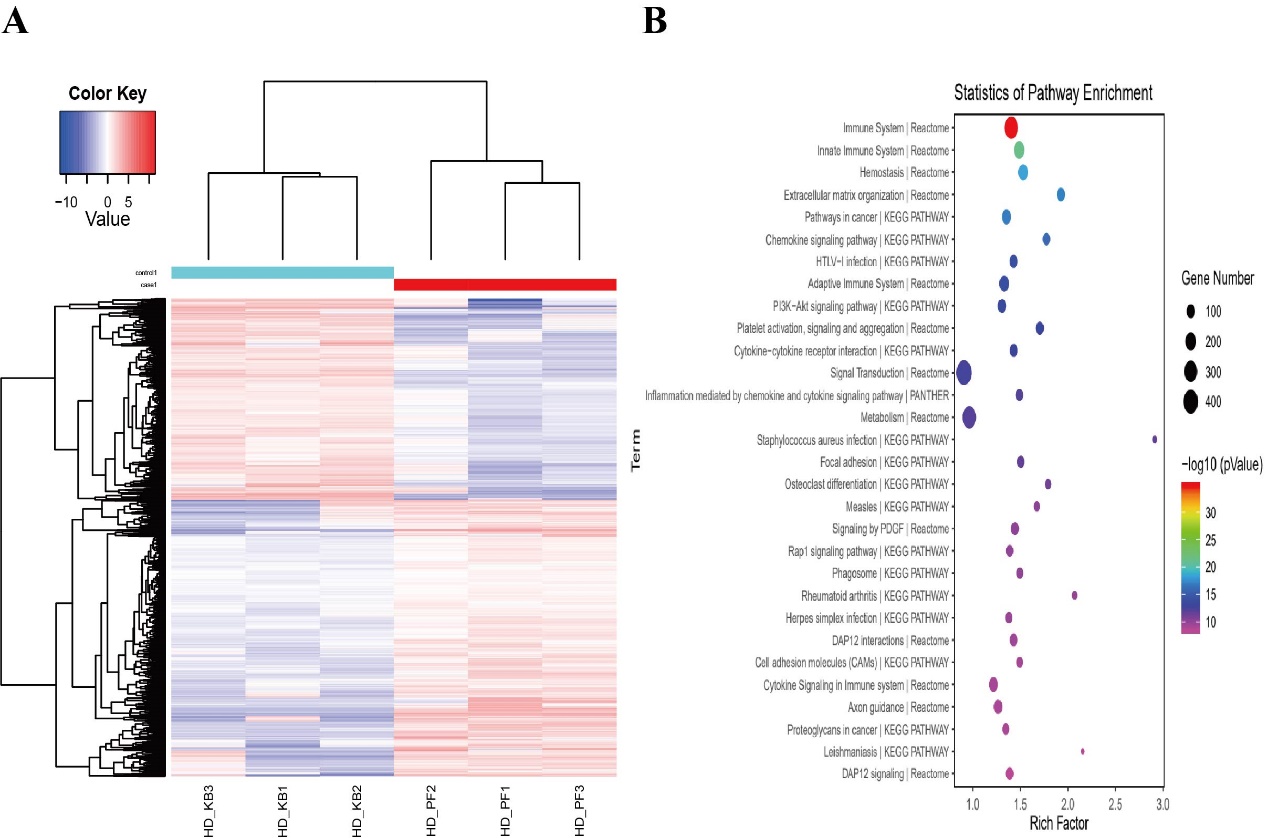
***

**Figure S2.** **Transcriptome data analysis.** (A) Cluster map of differential genes in mice treated with CEES. (B) Results of disease enrichment analysis of differential genes in CEES infected group.


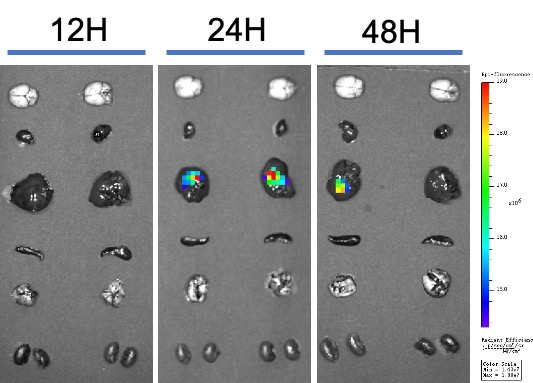


**Figure S3.** **the main live organs of live fluorescence images** of animals injected with Cy7-loaded microneedles.
